# Association of a Hospital-Wide Integrated Stewardship Intervention with Hospital-Acquired Multidrug-Resistant Organism Infection Incidence Density: A Large-Scale Interrupted Time-Series Study

**DOI:** 10.3390/antibiotics15050476

**Published:** 2026-05-07

**Authors:** Shan Zheng, Li Yang, Cong Shi, Chuan Xu, Li Tan

**Affiliations:** 1 Department of Hospital Infection Management, Tongji Hospital, Tongji Medical College, Huazhong University of Science and Technology, Wuhan 430030, China; m202476085@hust.edu.cn (S.Z.); liliyang2024@hust.edu.cn (L.Y.); 15587458931@163.com (C.S.); 2 School of Medicine and Health Management, Tongji Medical College, Huazhong University of Science and Technology, Wuhan 430030, China

**Keywords:** antimicrobial stewardship, drug resistance, microbial, cross infection, anti-bacterial agents, time series studies, hospitals, teaching

## Abstract

**Background**: Hospital-acquired multidrug-resistant organism (HA-MDRO) infections remain a major patient-safety threat linked to antimicrobial exposure, but long-term hospital-level evidence on whether integrated stewardship can reduce HA-MDRO burden remains limited. **Methods**: We conducted a quasi-experimental interrupted time-series study at a large multi-campus tertiary teaching hospital in China. A hospital-wide integrated intervention combining diagnostic stewardship and antimicrobial prescribing stewardship was implemented on 1 November 2021. Monthly aggregated hospital data from July 2018 to December 2024, including 2,145,489 hospitalizations, were analyzed. The primary outcome was HA-MDRO infection incidence density per 1000 patient-days. **Results**: HA-MDRO incidence density decreased immediately at the start of the COVID period (IRR = 0.246; *p* < 0.001) and then increased over time (IRR per month = 1.074; *p* < 0.001). After intervention implementation, the post-intervention trend declined significantly relative to the COVID-period trajectory (IRR per month = 0.938; *p* < 0.001). Microbiological testing increased immediately and continued to rise (OR = 1.381 and 1.016 per month, respectively), whereas restricted antibiotic use declined after implementation (OR = 0.979 per month; all *p* < 0.05). The control outcome showed no consistent post-intervention change. Counterfactual analysis estimated that 15,274 HA-MDRO cases were averted over follow-up. **Conclusions**: A hospital-wide integrated stewardship intervention was associated with reversal of the increasing HA-MDRO trajectory observed during the COVID period, together with improved microbiological testing and reduced restricted antibiotic use. These findings support the value of integrating diagnostic and prescribing stewardship in high-volume tertiary hospital settings.

## 1. Introduction

Hospital-acquired multidrug-resistant organism (HA-MDRO) infections remain a major threat to patient safety and clinical care worldwide and are increasingly encountered in intensive care settings, where they pose substantial challenges to infectious disease management [1,2]. Antimicrobial resistance was associated with an estimated 1.27 million deaths globally in 2019, and multidrug-resistant organisms may account for approximately one-fifth of healthcare-associated infections in hospitalized patients [3,4]. Compared with non-resistant infections, HA-MDRO infections are associated with greater treatment complexity, longer hospital stays, higher healthcare costs, and increased mortality risk [5]. Inappropriate antibiotic use, including unnecessarily broad-spectrum empirical therapy, redundant combination regimens, and prolonged courses without clear indications, increases selection pressure and accelerates the emergence and spread of antimicrobial resistance (AMR) [6,7]. Rising resistance further constrains effective therapy and may delay appropriate treatment, ultimately worsening clinical outcomes and increasing healthcare resource utilization [8]. Therefore, robust antimicrobial stewardship programs (ASPs) that optimize prescribing practices are essential to mitigate the growing burden of HA-MDRO infections and AMR [9].

Although ASPs have been widely implemented, important evidence gaps remain [10]. Many evaluations are from well-resourced hospitals in high-income settings, whereas evidence from low- and middle-income countries is limited [11,12]. In addition, studies often have short intervention periods and small sample sizes, limiting generalizability [13,14]. Prior evaluations have frequently focused on process and economic outcomes, such as prescribing appropriateness, antibiotic consumption, length of stay, and expenditures [15,16,17]. All-cause mortality is also commonly reported, but it is strongly influenced by competing factors and may be relatively insensitive to stewardship-related changes in infection risk [18]. By contrast, evidence on hospital-wide integrated stewardship interventions using HA-MDRO incidence as a clinically meaningful patient-safety outcome remains limited, particularly in high-volume, multi-campus tertiary hospital settings.

The Infectious Diseases Society of America emphasizes that the central goal of ASPs is to optimize clinical outcomes while minimizing unintended consequences of antimicrobial use, including toxicity and selection for resistant organisms [19]. In this context, HA-MDRO incidence is a clinically meaningful patient-safety outcome because it is more directly linked to antimicrobial exposure and local resistance ecology than broader endpoints such as all-cause mortality. Suboptimal empiric therapy, including delayed initiation, inadequate initial coverage, and unnecessarily broad-spectrum regimens, has been associated with worse outcomes and remains an important target for stewardship interventions [20,21]. However, prescribing oversight alone may be insufficient when microbiological evidence is delayed, unavailable, or not incorporated into clinical decision-making. Strengthening early microbiological diagnostic capacity and integrating diagnostic information into prescribing decisions may reduce uncertainty during empiric treatment, facilitate timely regimen optimization and de-escalation, and limit avoidable broad-spectrum exposure [22,23]. Accordingly, integrated strategies that combine diagnostic stewardship with prescribing interventions are increasingly recognized as an important component of hospital-wide ASPs.

Therefore, we conducted a quasi-experimental interrupted time-series study at a multi-campus tertiary teaching hospital in China. Using routinely collected monthly inpatient surveillance and electronic hospital data from July 2018 to December 2024, encompassing 2,145,489 hospitalizations, we evaluated the association between a hospital-wide integrated stewardship intervention implemented on 1 November 2021 and changes in HA-MDRO burden across the pre-COVID, COVID, and post-intervention periods. The intervention integrated diagnostic stewardship with antimicrobial prescribing stewardship to strengthen the timely acquisition and clinical use of microbiological data and to optimize antimicrobial use. The primary outcome was monthly HA-MDRO infection incidence density per 1000 patient-days. This study aimed to provide real-world evidence on the impact of hospital-wide integrated stewardship on HA-MDRO burden in a high-volume tertiary hospital setting.

## 2. Results

### 2.1. Study Population and Baseline Characteristics

A total of 2,145,489 hospitalizations were included across the three study periods: 330,943 pre-COVID, 572,165 during COVID, and 1,242,381 post-intervention. Table 1 summarizes patient population structure and aggregated clinical indicators across periods. Campus distribution differed significantly over time (*p* < 0.001), with admissions predominantly from the First Campus and an increased proportion from the Second Campus in the post-intervention period (23.08% vs. 13.99% pre-COVID and 14.54% during COVID). ICU status and department category also differed across periods (*p* = 0.038 and *p* < 0.001, respectively). HA-MDRO infection incidence density declined from 0.15 pre-COVID to 0.07 during COVID, then increased to 0.11 post-intervention (*p* < 0.001). The microbiological testing rate increased to 53.82% post-intervention, while the restricted antibiotic use rate decreased to 23.45% (both *p* < 0.001). Non-HA-MDRO infection incidence density was lower during COVID and post-intervention than in the pre-COVID period (*p* < 0.001).

### 2.2. Interrupted Time-Series Analysis of Primary, Secondary, and Control Outcomes

**Figure 1:**
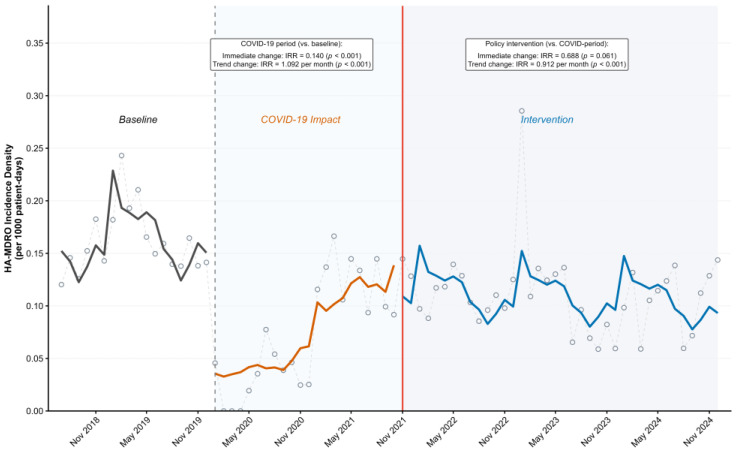
Interrupted time-series analysis of HA-MDRO infection incidence density (per 1000 patient-days) across the baseline, COVID-19, and post-intervention periods. Open circles indicate observed monthly values; colored solid lines indicate fitted trends for each period: black for the baseline period, orange for the COVID-19 period, and blue for the post-intervention period. Dashed vertical lines indicate period boundaries, and the red vertical line indicates the implementation of the intervention.

Table 2 and Figure 1 summarize the interrupted time-series results for HA-MDRO infection incidence density. Compared with the baseline period, the onset of the COVID-19 period was associated with a significant immediate decrease in HA-MDRO incidence density (IRR = 0.246; 95% CI: 0.130–0.464; *p* < 0.001), followed by a significant upward trend during the COVID period (IRR per month = 1.074; 95% CI: 1.038–1.111; *p* < 0.001). At the time of intervention implementation in November 2021, no significant immediate level change was observed relative to the COVID period (IRR = 0.784; 95% CI: 0.519–1.185; *p* = 0.248). However, the post-intervention trend declined significantly compared with the COVID-period trend (IRR per month = 0.938; 95% CI: 0.906–0.971; *p* < 0.001), indicating reversal of the previous increasing trajectory.

**Figure 2:**
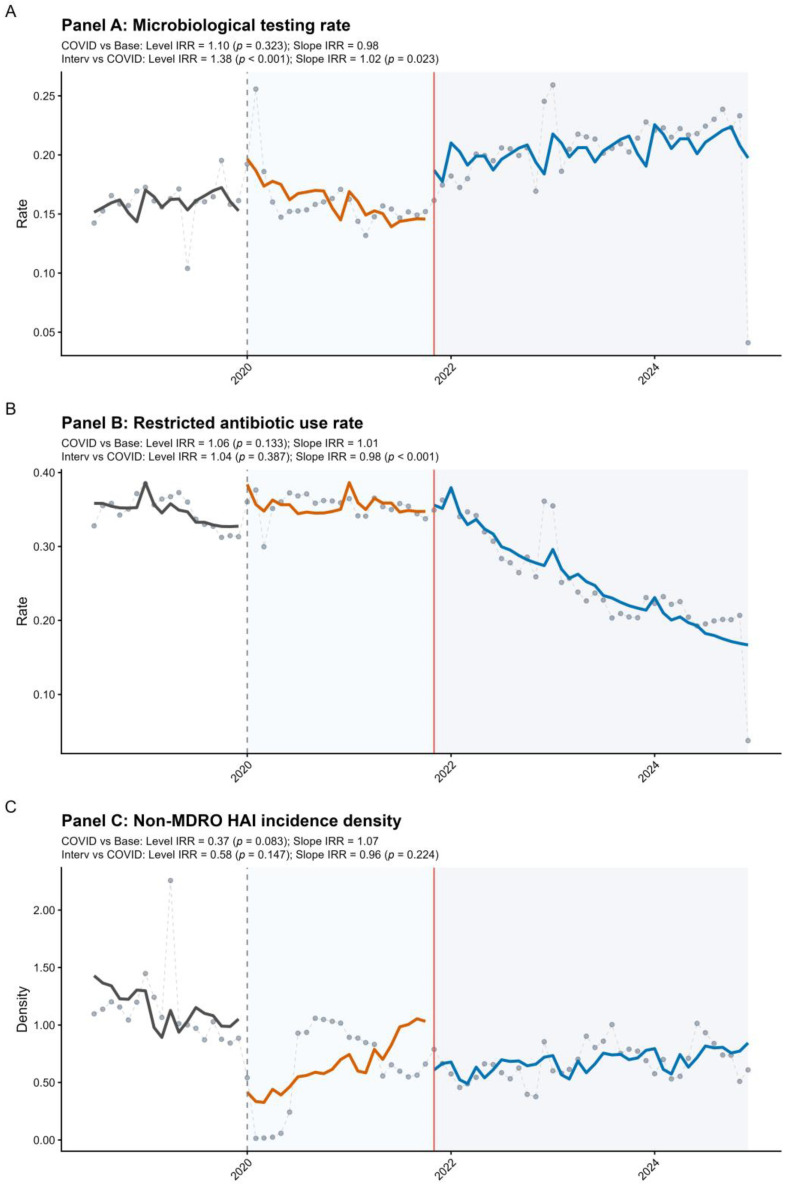
Interrupted time-series of secondary and control outcomes across the baseline, COVID-19, and post-intervention periods: (**A**) microbiological testing rate; (**B**) restricted antibiotic use rate; and (**C**) non-MDRO HAI incidence density. Open circles represent observed monthly values; black, orange, and blue lines represent fitted trends in the baseline, COVID-19, and post-intervention periods, respectively. Dashed vertical lines indicate period boundaries, and the red vertical line indicates intervention implementation.

Table 2 and Figure 2 summarize the ITS estimates and monthly trajectories for the secondary and control outcomes. For microbiological testing, no significant immediate level change was observed at the start of the COVID period (OR = 1.105; 95% CI: 0.907–1.346; *p* = 0.323), although the trend decreased slightly during that period (OR per month = 0.982; 95% CI: 0.969–0.996; *p* = 0.013). After intervention implementation, the testing rate increased immediately (OR = 1.381; 95% CI: 1.177–1.620; *p* < 0.001) and continued to increase over time (OR per month = 1.016; 95% CI: 1.002–1.030; *p* = 0.023). For restricted antibiotic use, neither the immediate level change at the start of the COVID period (OR = 1.063; 95% CI: 0.982–1.151; *p* = 0.133) nor that at intervention implementation (OR = 1.040; 95% CI: 0.951–1.138; *p* = 0.387) was significant; however, the trend increased slightly during the COVID period (OR per month = 1.007; 95% CI: 1.000–1.013; *p* = 0.048) and declined significantly after the intervention (OR per month = 0.979; 95% CI: 0.972–0.986; *p* < 0.001). For the control outcome, non-MDRO HAI incidence density showed no significant immediate or trend changes at either interruption point.

### 2.3. Sensitivity Analyses

Sensitivity analyses supported the main findings (Supplementary Tables S1–S3). For HA-MDRO incidence density, findings were consistent after Fourier seasonality adjustment, exclusion of the implementation transition period, exclusion of the COVID peak month, and autocorrelation-robust inference. The immediate level change was attenuated under the Newey–West HAC specification, but the post-intervention slope decrease remained significant across analyses. Results for microbiological testing were also robust, with significant post-intervention increases in both level and slope. Restricted antibiotic use showed no significant immediate level change, but the post-intervention slope decline was consistent in all models. For non-MDRO HAI incidence density, evidence for post-intervention change was limited.

### 2.4. Heterogeneity Analyses

Heterogeneity analyses of HA-MDRO infection incidence density showed limited evidence of effect modification in post-intervention slope changes (Supplementary Table S4). Across campuses, a significant post-intervention slope change was observed in the First Campus, whereas no significant change was detected in the Second Campus or Third Campus; however, interaction analyses did not support clear heterogeneity by campus. Similarly, the post-intervention slope change was significant in the non-ICU subgroup but not in the ICU subgroup, with no evidence of effect modification by ICU status. By department category, significant post-intervention slope changes were observed in the medical and surgical subgroups, whereas the estimate for other departments was not statistically significant. Interaction analysis suggested possible heterogeneity by department category, with a significant interaction for the surgical subgroup compared with the medical reference group (*p* = 0.046).

### 2.5. Counterfactual Averted HA-MDRO Infections

**Figure 3:**
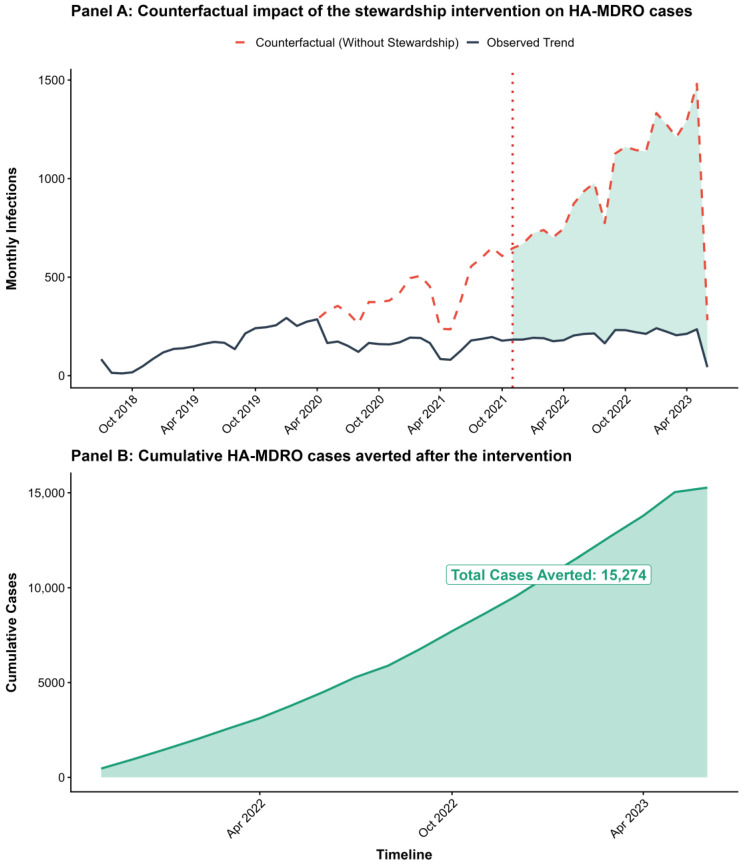
Counterfactual analysis of HA-MDRO cases. (**A**) Observed and counterfactual monthly cases after intervention implementation. The dark solid line represents the observed trend, the red dashed line represents the counterfactual trend without stewardship, and the red vertical dotted line indicates intervention implementation. The green shaded area represents estimated cases averted. (**B**) Cumulative cases averted after intervention implementation, shown by the green line and shaded area (total = 15,274).

Counterfactual analysis based on the segmented model suggested that, after accounting for baseline and COVID-period changes, HA-MDRO cases would have remained substantially higher under a no-policy scenario during the post-intervention period (Figure 3A). Following implementation in November 2021, observed monthly cases were lower than the counterfactual estimates in most months, and the shaded area represents the estimated number of cases averted by the intervention. By the end of follow-up, the intervention was estimated to have prevented 15,274 HA-MDRO cases. The cumulative number of averted cases increased steadily over time after implementation (Figure 3B).

### 2.6. Restricted Cubic Spline Analyses

**Figure 4:**
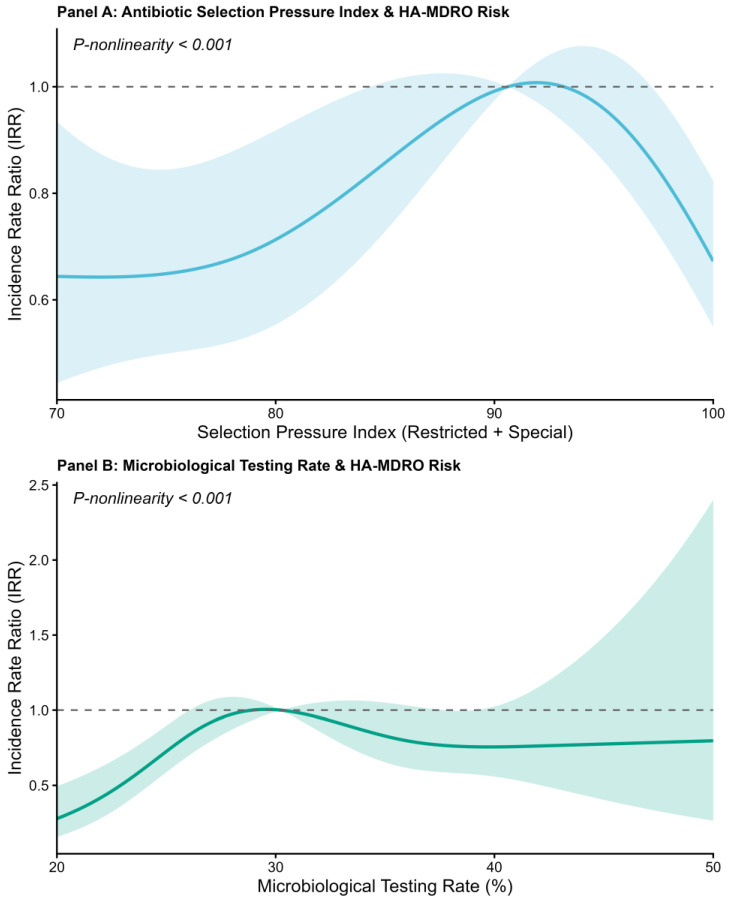
Restricted cubic spline analyses of HA-MDRO risk. (**A**) Antibiotic selection pressure index. (**B**) Microbiological testing rate. Solid lines represent estimated incidence rate ratios, shaded areas represent 95% confidence intervals, and dashed horizontal lines indicate the reference value of 1.0.

Restricted cubic spline analyses showed significant non-linear associations with HA-MDRO risk (*p* for non-linearity < 0.001 for both; Figure 4). For the antibiotic selection pressure index, the estimated HA-MDRO risk was relatively stable at lower levels, increased steadily as the index rose, peaked around 90–93%, and then declined at the highest values. For the microbiological testing rate, the estimated risk increased from low levels to approximately 30%, after which it decreased and remained relatively stable at a lower level across much of the higher testing range. Confidence intervals widened at the extremes of both distributions.

## 3. Discussion

This interrupted time-series study, encompassing more than 2.14 million hospitalizations over 6.5 years (July 2018 to December 2024) across the pre-COVID, COVID, and post-intervention periods, showed that a hospital-wide integrated stewardship intervention was associated with a clear reversal of the HA-MDRO trajectory. During the COVID period, HA-MDRO incidence density increased over time (IRR = 1.074), whereas after implementation in November 2021, the post-intervention trend declined significantly (IRR = 0.938). Counterfactual analysis further suggested that 15,274 HA-MDRO cases were averted over follow-up. These changes were accompanied by higher microbiological testing and lower restricted antibiotic use, supporting the value of an integrated diagnostic-prescribing stewardship strategy in high-volume tertiary hospital settings.

Timely microbiological testing and pathogen identification can reduce diagnostic uncertainty and support more appropriate empiric therapy [24,25,26]. In line with prior work, our hospital-wide integrated intervention was associated with a reversal of the increasing HA-MDRO trajectory after implementation [27]. The increase in microbiological testing occurred in parallel with the decline in infection risk, supporting synergy between diagnostic stewardship and prescribing oversight. Incorporating diagnostic results into prescribing may facilitate earlier regimen optimization and de-escalation, thereby limiting unnecessary broad-spectrum exposure [28,29]. Residual month-to-month fluctuations likely reflect changes in case mix and invasive device utilization [30].

Consistent with prior studies, restricted antibiotic use declined after implementation [31]. This pattern likely reflects the combined effects of prospective audit and feedback and intensified diagnostic stewardship. Pharmacist-led review may have improved adherence to prescribing indications and standardized regimen selection, thereby reducing avoidable exposure to higher-tier agents [32]. In parallel, increased microbiological testing provided timely etiological evidence to support earlier regimen optimization and de-escalation, shortening unnecessary broad-spectrum therapy and promoting more targeted treatment [33].

Restricted cubic spline analyses showed non-linear associations between the antibiotic selection pressure index and HA-MDRO risk. The estimated risk remained relatively stable at lower levels, increased progressively across a higher range, and declined at the highest levels. This pattern may reflect differences in case mix across selection-pressure strata and concurrent management responses. Higher use of high-grade antibiotics likely tracks greater illness severity and baseline resistance burden, whereas intensified audit and feedback, de-escalation, and infection prevention at higher selection pressure may contribute to the subsequent risk decline [34,35,36,37]. Microbiological testing rate also showed a non-linear association with HA-MDRO risk: risk increased at lower testing levels, then decreased across a wider range of higher testing rates. Early increases in testing may raise case ascertainment, while more comprehensive testing can provide timely evidence to support de-escalation and reduce unnecessary broad-spectrum exposure [38,39].

The inclusion of non-MDRO HAI incidence density as a control outcome provides important context for interpreting the main findings. In contrast to the clearer post-intervention pattern observed for HA-MDRO incidence density, non-MDRO HAI showed no consistent post-intervention trend change (*p* = 0.224). This divergence suggests that the reduction in HA-MDRO burden was unlikely to reflect a uniform shift across all hospital-acquired infections during the study period. Because the intervention specifically targeted diagnostic and antimicrobial pathways, the absence of a parallel decline in the control outcome supports the specificity of the observed association, although residual confounding from unmeasured time-varying factors cannot be fully excluded.

At the departmental level, the observed heterogeneity may reflect differences in baseline diagnostic workflow and prescribing structure between surgical and medical services. Surgical units often face greater diagnostic uncertainty at antibiotic initiation and may therefore rely more heavily on empirical broad-spectrum treatment when microbiological evidence is limited. In our study, lower baseline microbiological testing and higher restricted antibiotic use in surgical services suggest greater room for optimization, which may explain the larger post-intervention gains after diagnostic stewardship and audit-and-feedback were strengthened [40]. By contrast, internal medicine may have had more standardized baseline testing and antimicrobial decision-making, limiting the magnitude of further improvement after implementation [41,42,43].

This study has several limitations. First, as a single-center retrospective study, the generalizability of our findings to other hospitals, healthcare systems, or stewardship settings may be limited. Second, although the interrupted time-series design, double-breakpoint modeling, control outcome analysis, and multiple sensitivity analyses strengthened internal validity, this quasi-experimental approach cannot fully establish causality, and residual confounding from unmeasured time-varying factors remains possible. In particular, changes in case mix, healthcare utilization, infection prevention practices, diagnostic intensity, and secular trends during and after the COVID-19 period may have influenced the observed associations. Third, the use of monthly aggregated hospital-level data precluded patient-level assessment of antibiotic exposure, microbiological testing, clinical severity, device use, and individual infection risk. Finally, because the intervention was implemented as a hospital-wide integrated strategy, the separate contributions of its diagnostic and prescribing components could not be disentangled.

## 4. Materials and Methods

### 4.1. Study Design and Setting

We conducted a single-center, multi-campus, quasi-experimental interrupted time-series study using monthly aggregated hospital-level data to assess changes in HA-MDRO infection incidence density associated with a hospital-wide integrated stewardship intervention. The study was performed at a tertiary teaching hospital in Wuhan, China, with approximately 6000 inpatient beds across multiple campuses. The study period was July 2018 to December 2024, including a pre-COVID period (July 2018–December 2019), a COVID/pre-intervention period (January 2020–October 2021), and a post-intervention period (November 2021–December 2024). The intervention was implemented on 1 November 2021. This study was reported with reference to the STROBE statement and methodological guidance for interrupted time-series analyses. No formal sample size calculation was performed because all available routine hospital data were included. Ethical approval was obtained from the Ethics Committee of Tongji Hospital, Tongji Medical College, Huazhong University of Science and Technology on 27 October 2025 (TJ-IRB202510077), with waiver of informed consent.

### 4.2. Intervention

A hospital-wide integrated stewardship intervention was implemented on 1 November 2021 across all inpatient services and campuses. Rather than relying on educational or administrative measures alone, the intervention established a technology-enabled “diagnostic-prescribing” closed-loop workflow coordinated by the Infection Prevention and Control Department, pharmacy department, clinical microbiology laboratory, and hospital information department. Diagnostic stewardship promoted microbiological testing before therapeutic antibiotic initiation through hospital targets, standardized specimen management, laboratory quality control, staff training, and routine feedback, supported by electronic prompts and automated indicator capture in the hospital information system. In parallel, prescribing stewardship targeted restricted- and special-grade antibiotics through pharmacist-led prospective audit and feedback and department-level review of higher-tier and combination therapy use. All core components were introduced concurrently and sustained through continuous monthly monitoring during the post-intervention period.

### 4.3. Data Sources

We used de-identified monthly aggregated hospital-level data from routine electronic information systems of Tongji Hospital, including administrative admission records, inpatient antimicrobial prescribing records, microbiological testing and susceptibility data, and healthcare-associated infection (HAI) surveillance records. All inpatient admissions from all campuses during July 2018 to December 2024 were included; outpatient and emergency encounters were excluded. For antibiotic-related indicators, perioperative prophylaxis was excluded and only therapeutic antibiotic use was analyzed; restricted- and special-grade agents were classified according to the national antibiotic management catalogue [44]. HAIs were identified through routine surveillance using China’s national diagnostic criteria, and surveillance procedures remained unchanged during the study period. HA-MDRO infections were ascertained by linking adjudicated HAI events with microbiological susceptibility results. Newly identified HA-MDRO episodes were counted once in the monthly series to avoid duplicate counting. Monthly indicators were generated after consistency checks across source systems, and no formal imputation was required.

### 4.4. Outcomes

The primary outcome was monthly HA-MDRO infection incidence density, defined as the number of newly identified HA-MDRO infections per 1000 patient-days [27]. Secondary outcomes were the monthly microbiological testing rate, defined as the proportion of hospitalizations receiving therapeutic antibiotics in which microbiological testing was performed within 24 h before or after antibiotic initiation [45], and restricted antibiotic use rate, defined as the proportion of hospitalizations with receipt of at least one restricted-grade systemic antibiotic according to the national catalogue [46]. Non-MDRO HAI incidence density was analyzed as a control outcome and defined as the number of HAIs not attributed to MDROs per 1000 patient-days. For restricted cubic spline analyses, the antibiotic selection pressure index was defined as the combined proportion of restricted- and special-grade antibiotic use among hospitalizations receiving therapeutic antibiotics [47].

### 4.5. Statistical Analysis

Interrupted time-series analyses were conducted using monthly aggregated hospital-level data. Monthly HA-MDRO counts were modeled using segmented negative binomial regression with log(patient-days) as an offset. Models included level and slope changes at the onset of the COVID period (January 2020) and at intervention implementation (November 2021). Secondary proportion outcomes were analyzed using segmented generalized linear models, and non-MDRO HAI incidence density was analyzed as a control outcome using the same segmented framework as the primary outcome. Effect estimates are reported as IRRs or ORs with 95% confidence intervals. Seasonality and autocorrelation were assessed a priori and examined through month terms, Fourier adjustment, and autocorrelation-robust inference. Sensitivity analyses included Fourier adjustment, exclusion of the intervention transition period, exclusion of the COVID peak month, and Newey–West robust standard errors. Heterogeneity analyses by campus, ICU status, and department category were exploratory. Counterfactual averted cases were estimated by projecting the no-intervention trajectory into the post-intervention period. Restricted cubic spline models with four knots examined non-linear associations of the antibiotic selection pressure index and microbiological testing rate with HA-MDRO risk. Analyses were performed in R (version 4.4.3; R Foundation for Statistical Computing, Vienna, Austria), with two-sided *p* < 0.05 considered statistically significant.

## 5. Conclusions

This interrupted time-series study of 2,145,489 hospitalizations showed that a hospital-wide integrated intervention combining diagnostic and antimicrobial stewardship was associated with reversal of the increasing HA-MDRO trajectory observed during the COVID period. This change was accompanied by increased microbiological testing, reduced restricted antibiotic use, and an estimated 15,274 HA-MDRO cases averted over follow-up. The lack of a consistent post-intervention change in the control outcome supports the specificity of the observed association. These findings highlight the value of integrated diagnostic and prescribing stewardship in tertiary hospital settings.

## Figures and Tables

**Table 1:** Characteristics and clinical indicators across three study periods.

Characteristics and Outcomes	Pre-COVID (*N* = 330,943)	During-COVID (*N* = 572,165)	Post-Intervention (*N* = 1,242,381)	*p* Value
**Volume**				
Total admissions	330,943	572,165	1,242,381	—
Total patient-days	2,415,884	4,119,588	8,845,142	<0.001
Median LOS (IQR), days	7.30 [4.00, 9.00]	7.20 [4.0, 10.00]	7.10 [4.00, 9.00]	<0.001
**Patient population structure, *n* (%)**				
Campus				<0.001
First campus	225,481 (68.12%)	384,619 (67.22%)	744,528 (59.93%)	
Second campus	46,312 (13.99%)	83,186 (14.54%)	286,750 (23.08%)	
Third campus	59,240 (17.89%)	104,360 (18.24%)	211,103 (16.99%)	
ICU status				0.038
Non-ICU	328,064 (99.13%)	567,162 (99.13%)	1,229,113 (98.93%)	
ICU	2879 (0.87%)	5003 (0.87%)	13,268 (1.07%)	
Department category				<0.001
Medical	150,211 (45.39%)	262,947 (45.96%)	525,410 (42.29%)	
Surgical	135,112 (40.83%)	231,720 (40.50%)	505,446 (40.68%)	
Others	45,620 (13.78%)	77,498 (13.54%)	211,525 (17.03%)	
**Clinical indicators, median [IQR]**				
HA-MDRO infection incidence density^1^	0.15 [0.14, 0.18]	0.07 [0.03, 0.11]	0.11 [0.09, 0.13]	<0.001
Microbiological testing rate (%)	37.62 [36.50, 38.40]	37.17 [35.30, 38.20]	53.82 [47.90, 56.40]	<0.001
Restricted antibiotic use rate (%)	35.29 [32.80, 36.30]	35.88 [35.00, 36.40]	23.45 [20.50, 30.20]	<0.001
Non-HA-MDRO infection incidence density ^1^	1.05 [0.98, 1.19]	0.66 [0.54, 0.92]	0.66 [0.58, 0.78]	<0.001

Abbreviations: IQR, interquartile range; LOS, length of stay; HA-MDRO, hospital-acquired multidrug-resistant organism;. Notes: Data are presented as n (%), median [IQR], or total counts, as appropriate. *p* values were derived from one-way ANOVA, Kruskal-Wallis test, or Pearson’s chi-square test, as appropriate. ^1^ Density indicators are expressed as episodes per 1000 patient-days.

**Table 2:** Interrupted time-series regression results across study outcomes.

Outcome	Term	Effect (IRR/OR)	95% CI	*p* Value
**HA-MDRO infection** **incidence density**	COVID-period level change	0.246	(0.130, 0.464)	<0.001
	COVID-period trend change	1.074	(1.038, 1.111)	<0.001
	Post-intervention level change	0.784	(0.519, 1.185)	0.248
	Post-intervention trend change	0.938	(0.906, 0.971)	<0.001
**Microbiological testing rate**	COVID-period level change	1.105	(0.907, 1.346)	0.323
	COVID-period trend change	0.982	(0.969, 0.996)	0.013
	Post-intervention level change	1.381	(1.177, 1.620)	<0.001
	Post-intervention trend change	1.016	(1.002, 1.030)	0.023
**Restricted antibiotic use rate**	COVID-period level change	1.063	(0.982, 1.151)	0.133
	COVID-period trend change	1.007	(1.000, 1.013)	0.048
	Post-intervention level change	1.04	(0.951, 1.138)	0.387
	Post-intervention trend change	0.979	(0.972, 0.986)	<0.001
**Non-MDRO HAI** **incidence density**	COVID-period level change	0.372	(0.122, 1.137)	0.083
	COVID-period trend change	1.069	(0.991, 1.153)	0.085
	Post-intervention level change	0.578	(0.275, 1.213)	0.147
	Post-intervention trend change	0.959	(0.896, 1.026)	0.224

Effects are presented as incidence rate ratios (IRRs) for incidence density outcomes and odds ratios (ORs) for rate outcomes. COVID-period effects are relative to the baseline period, and post-intervention effects are relative to the COVID period. Values are shown with 95% confidence intervals.

## Data Availability

The data are not publicly available due to privacy restrictions; data may be available from the corresponding authors upon reasonable request.

## Supplementary Materials

The following supporting information can be downloaded at: https://www.mdpi.com/article/10.3390/antibiotics15050476/s1. Table S1. Sensitivity analyses for HA-MDRO incidence density; Table S2. Sensitivity analyses for microbiological testing rate; Table S3. Sensitivity analyses for restricted antibiotic use rate; Table S4. Heterogeneity analyses of post-intervention slope change in HA-MDRO infection incidence density.

## Abbreviations

The following abbreviations are used in this manuscript:

HA-MDRO	Hospital-acquired multidrug-resistant organism
ITS	Interrupted time-series
AMR	Antimicrobial resistance
