# Supplementary material for: Association of a Hospital-Wide Integrated Stewardship Intervention with Hospital-Acquired Multidrug-Resistant Organism Infection Incidence Density: A Large-Scale Interrupted Time-Series Study
Source: Antibiotics (Basel). 2026 May 7;15(5):476. doi: 10.3390/antibiotics15050476 (PMC13203878; doi:10.3390/antibiotics15050476)
Supplement: Supplementary file 1 [file antibiotics-15-00476-s001.zip › antibiotics-4214676-supplementary.pdf]

Table S1. Sensitivity analyses for HA-MDRO infection incidence density

Model specification	Effect	IRR	95% CI	P value
1. Main model	Level change	0.688	(0.499, 0.950)	0.023
	Slope change	0.912	(0.883, 0.943)	<0.001
2. Fourier seasonality	Level change	0.711	(0.517, 0.977)	0.036
	Slope change	0.914	(0.885, 0.945)	<0.001
3. Wash-out period	Level change	0.629	(0.454, 0.870)	0.005
	Slope change	0.916	(0.887, 0.946)	<0.001
4. Excluding the COVID-19 peak	Level change	0.704	(0.512, 0.966)	0.030
	Slope change	0.916	(0.888, 0.946)	<0.001
5. Newey–West HAC	Level change	0.688	(0.466, 1.017)	0.061
	Slope change	0.912	(0.881, 0.945)	<0.001

Footnote

IRRs were estimated from interrupted time-series models. Level change indicates the immediate change at intervention implementation, and slope change indicates the change in post-intervention trend.

Table S2. Sensitivity analyses for microbiological testing rate

Model specification	Effect	OR	95% CI	P value
1. Main model (HC1)	Level change	1.381	(1.187, 1.607)	<0.001
	Slope change	1.016	(1.003, 1.029)	0.016
2. Fourier adjustment	Level change	1.38	(1.181, 1.613)	<0.001
	Slope change	1.016	(1.004, 1.029)	0.011
3. Wash-out period	Level change	1.411	(1.212, 1.643)	<0.001
	Slope change	1.015	(1.002, 1.028)	0.023
4. Newey–West HAC	Level change	1.381	(1.177, 1.620)	<0.001
	Slope change	1.016	(1.002, 1.030)	0.023

Footnote

ORs were estimated from interrupted time-series models. Level change indicates the immediate change at intervention implementation, and slope change indicates the change in post-intervention trend.

Table S3. Sensitivity analyses for restricted antibiotic use rate

Model specification	Effect	OR	95% CI	P value
1. Main model (HC1)	Level change	1.04	(0.946, 1.144)	0.415
	Slope change	0.979	(0.972, 0.986)	<0.001
2. Fourier adjustment	Level change	1.045	(0.955, 1.143)	0.337
	Slope change	0.979	(0.973, 0.986)	<0.001
3. Wash-out period	Level change	1.038	(0.933, 1.154)	0.493
	Slope change	0.979	(0.972, 0.986)	<0.001
4. Newey–West HAC	Level change	1.04	(0.951, 1.138)	0.387
	Slope change	0.979	(0.972, 0.986)	<0.001

Footnote

ORs were estimated from interrupted time-series models. Level change indicates the immediate change at intervention implementation, and slope change indicates the change in post-intervention trend.

Table S4. Heterogeneity analyses of post-intervention slope change in HA-MDRO infection incidence density

Dimension	Group	IRR (95% CI)	P value	Interaction term	P for interaction
Campus	First campus	1.022 (1.006–1.039)	0.007	Reference	—
	Second campus	0.999 (0.987–1.012)	0.878	1.001 (0.991–1.011)	0.855
	Third campus	1.002 (0.978–1.028)	0.859	0.991 (0.981–1.001)	0.07
ICU status	ICU	0.997 (0.984–1.009)	0.581	Reference	—
	Non-ICU	1.015 (1.002–1.029)	0.028	1.004 (0.994–1.013)	0.457
Department category	Medical	1.017 (1.004–1.030)	0.009	Reference	—
	Others	0.983 (0.966–1.001)	0.068	0.991 (0.981–1.001)	0.083
	Surgical	1.029 (1.016–1.041)	<0.001	1.011 (1.000–1.022)	0.046

Footnote

Values are presented as incidence rate ratios (IRRs) with 95% confidence intervals for post-intervention slope change in HA-MDRO infection incidence density. Subgroup-specific estimates were obtained from stratified models. Interaction terms represent the relative difference in post-intervention slope change compared with the reference group within each dimension.
